# Depigmented Allergoids Reveal New Epitopes with Capacity to Induce IgG Blocking Antibodies

**DOI:** 10.1155/2013/284615

**Published:** 2013-10-08

**Authors:** M. Angeles López-Matas, Mayte Gallego, Víctor Iraola, Douglas Robinson, Jerónimo Carnés

**Affiliations:** R&D Department, Laboratorios LETI S.L., Calle del Sol 5, Tres Cantos, 28760 Madrid, Spain

## Abstract

*Background*. The synthesis of allergen-specific blocking IgGs that interact with IgE after allergen immunotherapy (SIT) has been related to clinical efficacy. The objectives were to investigate the epitope specificity of IgG-antibodies induced by depigmented-polymerized (Dpg-Pol) allergoids and unmodified allergen extracts, and examine IgE-blocking activity of induced IgG-antibodies. *Methods*. Rabbits were immunized with native and Dpg-Pol extracts of birch pollen, and serum samples were obtained. Recognition of linear IgG-epitopes of Bet v 1 and Bet v 2 and the capacity of these IgG-antibodies to block binding of human-IgE was determined. *Results*. Serum from rabbits immunized with native extracts recognised 11 linear epitopes from Bet v 1, while that from Dpg-Pol-immunized animals recognised 8. For Bet v 2, 8 epitopes were recognized by IgG from native immunized animals, and 9 from Dpg-Pol immunized one. Dpg-Pol and native immunized serum did not always recognise the same epitopes, but specific-IgG from both could block human-IgE binding sites for native extract. *Conclusions*. Depigmented-polymerized birch extract stimulates the synthesis of specific IgG-antibodies which recognize common but also novel epitopes compared with native extracts. IgG-antibodies induced by Dpg-Pol effectively inhibit human-IgE binding to allergens which may be part of the mechanism of action of SIT.

## 1. Introduction

Specific allergen immunotherapy (SIT) to the offending allergens confers clinical benefits in patients suffering from allergic diseases and is the only effective treatment with capacity to modify the evolution of the disease. Different markers have been suggested over the years to measure the immunological effect of the vaccines and to predict clinical benefits. Whilst no marker is yet sufficiently validated for routine clinical monitoring of SIT, induction of allergen-specific IgG antibodies (mainly IgG4 subclass) with the capacity to block allergen-IgE interaction has long been suggested as a potential mechanism for SIT [1] and more recently related to long-term benefit [2]. Other parameters related to cellular response, including T cell populations, regulatory T cells, or specific markers such as interleukin-10, which may be linked to switching to IgG4 production, are also considered as important and potential markers of response [3]. However, little is known about the process and mechanisms of IgG mediated inhibition in allergic reactions, and the concepts are not well defined. 

Mapping sequential and conformational IgG epitopes is becoming a potent tool for the exploration of the immunological response to allergens. However, little is known about the importance of such epitopes and their effect from an immunological point of view [4]. In the case of allergens, different studies support the concept that IgE epitopes are mainly conformational though the mapping of this kind of IgE epitopes remains a difficult task [5]. Recently, a new approach using human monoclonal IgE has been used to describe an IgE epitope to Bet v 1 [6].

Depigmented allergoids are allergenic extracts subjected to a mild acid treatment following by a polymerization with glutaraldehyde in order to reduce the allergenicity while preserving their immunogenicity. The resulting product is a molecule containing individual allergens in chains which have a high molecular weight. The clinical efficacy of depigmented allergoids has been clearly demonstrated in double blind placebo controlled clinical trials by reduction in symptoms and medication scores [7–10]. More recently these studies have been extended to show induction of specific IgG and IgG4 [11–13]. Using birch and grass pollen extracts, we demonstrated that depigmented allergoids induce an IgG immune response *in vivo* against individual allergens [14] and their corresponding isoforms [15]. Few data are available with other nondepigmented allergoids [16]. 

The objectives of this study were to investigate the epitope specificity of the antibody response induced in rabbits after immunization with depigmented-polymerized (Dpg-Pol) allergenic extracts of birch, using native extracts as control, and to determine the capacity of the generated IgG antibodies to block the allergen epitopes recognized by human IgE antibodies.

## 2. Materials and Methods

### 2.1. Extract Manufacturing

A native extract from birch pollen (Laboratorios LETI, Madrid, Spain) and its corresponding Dpg-Pol extract were manufactured following previously described methods. Briefly, 100 g of defatted *Betula alba* (=*pendula, verrucosa*) pollen (Allergon, Ängelhom, Sweden) was extracted in phosphate-buffered saline (PBS), 0.01 M pH 7.4. After centrifugation, the supernatant was collected, sterile filtered, dialyzed against highly purified water, filtered, frozen, and lyophilised. Lyophilised native extract was reconstituted in highly purified water and the pH reduced using a mild acid treatment in order to separate the low molecular weight substances. The extract was dialyzed in membranes with a cut-off of 3.5 kDa (Cellu Sep Membrane, Seguin, TX, USA) and all this material removed. The pH was again adjusted to physiological conditions, and the extract was sterile filtered, frozen, and lyophilised. Depigmented extract was reconstituted in PBS 0.01 M pH 7.4 and polymerised with glutaraldehyde. The resulting material was then dialyzed in 100 kDa dialysis membranes (Millipore, Bedford, USA), filtered, and lyophilised. Finally native and Dpg-Pol extracts were individually dissolved (260 *μ*g of protein/mL) and adsorbed to aluminum hydroxide (Brenntag, Mulheim, Germany).

### 2.2. Rabbit Immunization

Two New Zealand white rabbits were immunized with native and two with Dpg-Pol allergen extracts of *B. alba *adsorbed onto aluminum hydroxide (3%). Rabbits were immunized with 3 subcutaneous injections with 130 *μ*g of protein per dose, containing 15 *μ*g and 23 *μ*g of Bet v 1 for native and Dpg-Pol extract, respectively, following a previously described methodology [15]. Major allergen content was measured in the extract before polymerization using a commercial ELISA kit (Indoor Biotechnologies, VA, USA) as the guideline on production and quality of allergen products indicates [17]. 

Preimmune serum was immediately collected before the first immunization. Twenty-one days after the first immunization booster 1 was administered. Booster 2 was administered 51 days after the first immunization. Rabbits were bled after 71 days and the serum samples collected. Individual and pooled sera from both rabbits immunized with each extract were kept at −20°C. The rabbit immunization was conducted at the Vivotecnia Research facilities (Madrid, Spain). All the procedures were approved by the Institutional Review Board of Vivotecnia Research and followed the local ethical rules for animal experimentation.

### 2.3. Human Pool of Sera

Specific pool of sera was prepared after mixing the same quantity of 15 individual serum samples from allergic patients, clinically diagnosed as allergic to birch and with positive specific IgE to birch (78.9 kUA/l), rBet v 1 (95.6 kUA/l), and rBet v 2 (2.37 kUA/l). Serum samples were acquired from Plasmalab International (Everett, WA, USA) which operates in full compliance of Food and Drug Administration regulations. All serum samples were previously tested for carbohydrates recognition by CAP (Thermo Scientific, Uppsala, Sweden), using bromelin (nAna c 2) in solid phase. In all cases this assay was negative.

### 2.4. Immunological Response Characterization: IgG Titration

Specific IgG to birch, Bet v 1, and Bet v 2 were measured in serum samples from rabbits immunized with native and Dpg-Pol extracts by direct ELISA. Briefly, microplates (Nunc Maxisorp, Rosklide, Denmark) were coated with native *B. alba *extract (20 *μ*g/mL), rBet v 1, and rBet v 2 (1 *μ*g/mL) (Indoor Biotechnologies, VA, USA). Serum samples were diluted from 1 : 5,000 to 1 : 320,000 in serial dilutions and incubated for 2 hours. After washing, the secondary antibody consisting of Goat anti-Rabbit-IgG-Peroxidase (diluted 1 : 30,000) (Nordic Immunological Laboratories, Eindhoven, The Netherlands) was added. Finally microplates were washed, the reaction developed, and microplates read at 450 nm. Two independent experiments were performed, and measures were done in duplicate.

### 2.5. Recognition of Linear Epitopes of Bet v 1 and Bet v 2

Sequences of Bet v 1 (P15494) and Bet v 2 (P25816) were obtained from the UniProtKB/Swiss-Prot data bank (http://www.uniprot.org/). Linear synthetic peptides covalently bound to a cellulose membrane by the C-terminus (SPOTs) were commercially obtained from Sigma Genosys (JPT Peptide Technologies, Berlin, Germany). A total of 25 peptides containing 12 aminoacids and 1 containing 10 aminoacids were prepared with Bet v 1, and 24 peptides containing 12 aminoacids and 1 containing 13 aminoacids were prepared with Bet v 2. All of them were synthetized overlapping by 6 aminoacids. Synthetic peptides are shown in Table 1.

Membranes were rinsed with methanol for 5 minutes and washed with TRIS buffered saline (TBS) for 10 minutes. Afterwards they were blocked with blocking buffer (5% bovine serum albumin in TBS) for 2 hours. After washing, pool of sera obtained from native or Dpg-Pol immunized rabbits was individually incubated (dil 1 : 5,000) overnight. After the incubation with the secondary antibody, Goat anti-Rabbit-IgG-PO (Nordic Immunology), membranes were finally developed with RapidStep ECL Reagent (Merck Millipore, Darmstadt, Germany) and peptides identified by chemoluminescence with ChemiDoc XRS (BioRad Laboratories, Hercules, CA, USA).

### 2.6. IgG Inhibition

Microplates were coated with native and Dpg-Pol extracts (20 *μ*g/mL) and incubated at room temperature overnight. Sera from native and Dpg-Pol immunized rabbits were incubated with serial dilutions of native and Dpg-Pol extracts. After 2 hours, inhibited samples were incubated with coated microplates for 2 hours. After washing Goat anti-Rabbit-IgG-PO (1 : 30,000) (Nordic Immunology) was added and finally developed and read at 450 nm.

### 2.7. Immunoblot Inhibition

Native extract was electrophoretically (SDS-PAGE) separated and electrotransferred onto a P-Immobilon membrane (Millipore, Bedford, USA). A total of 65 *μ*g was loaded in each lane. Membranes were blocked for 2 hours with PBS 0.05% tween-1% skimmed milk. Serum samples from immunized rabbits (2 with native and 2 with Dpg-Pol extracts) were independently incubated at a dilution 1/400 for 2 hours with native birch extract (650 *μ*g). Afterwards inhibited serum samples were incubated with membranes overnight. After washing, membranes were incubated with anti-Rabbit-IgG-PO (1 : 30,000) (Nordic Immunology) and finally developed. Native serum samples without inhibition and those inhibited with bovine serum albumin (BSA) were used as controls.

### 2.8. Inhibition of Allergic Patients IgE Binding Proteins by Induced Specific IgG

The ability of native and Dpg-Pol extracts to induce IgG antibodies with capacity for blocking IgE epitopes of birch extract and rBet v 1 was investigated by ELISA inhibition experiments.

Briefly, microplates (Nunc Maxisorp) were coated, respectively, with native *B. alba *extract (20 *μ*g/mL) and rBet v 1 (1 *μ*g/mL) (Indoor Biotechnologies), blocked with 1% BSA-PBS-T, and incubated with native and Dpg-Pol rabbit sera and their preimmune sera. After washing, plates were incubated with the human pool of sera (1/10 dilution). Bound IgE was detected with anti-human-IgE-PO (Ingenasa, Madrid, Spain). Optical densities were measured at 450 nm.

Percentage of inhibition was calculated as follows: percentage of IgE binding = 100 − (OD_*i*_/OD_*P*_) × 100. OD_*i*_ and OD_*P*_ correspond to the optical densities after preincubation with the rabbit's immune sera and the corresponding preimmune sera, respectively [18].

## 3. Results

### 3.1. Induction of Specific IgG

**Figure 1:**
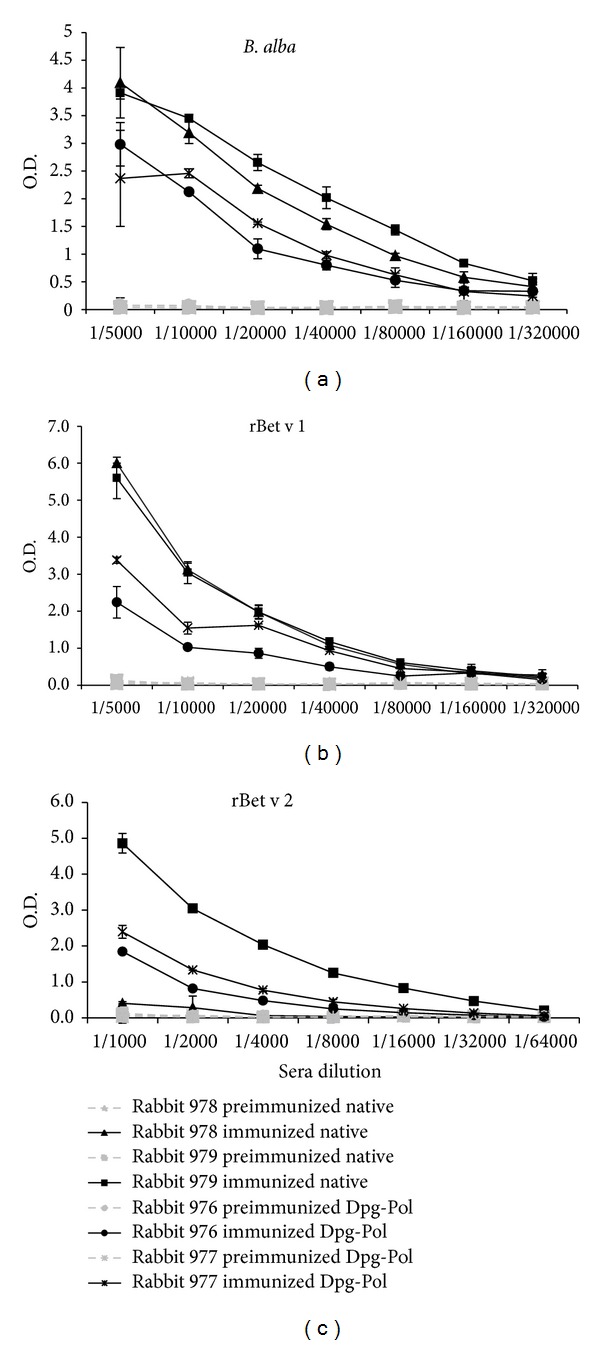
Titration of specific IgG antibodies from rabbits immunized with native or Dpg-Pol extracts of *B. alba*. Two rabbits were used for each extract. *B. alba *native extract, rBet v 1, and rBet v 2 specific IgG are shown. (a) Solid phase native *B. alba *extract, (b) solid phase rBet v 1, and (c) solid phase rBet v 2.

Both groups of rabbits immunized with native (rabbit 978 and 979) and Dpg-Pol (rabbit 976 and 977) extracts showed high titres of specific IgG antibodies against native extract and Bet v 1 and Bet v 2 purified allergens (Figure 1). Specific IgG values against the preimmune sera were negative in all rabbits.

### 3.2. Recognition of Linear Epitopes

**Figure 2:**
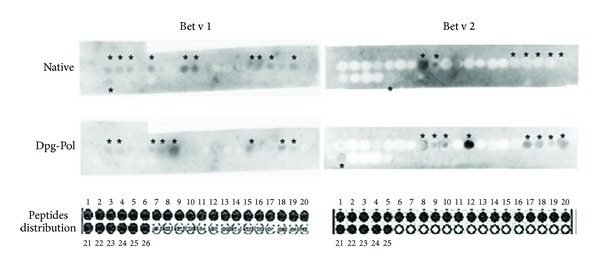
IgG linear epitopes from Bet v 1 and Bet v 2 recognized by rabbit sera immunized with native *B. alba* extract or Dpg-Pol extract. Recognized epitopes were marked with*. Distribution of epitopes in the membranes are shown.

Serum samples obtained from rabbits immunized with native and Dpg-Pol extracts recognised different IgG linear epitopes (Figure 2).

With respect to Bet v 1, serum samples from rabbits immunized with native extracts recognised 11 epitopes while serum samples from Dpg-Pol immunized animals recognised 8 epitopes. In case of Bet v 2, 8 epitopes were recognized from animals immunized with native extracts and 9 epitopes from Dpg-Pol immunized animals. 

Summarizing, Dpg-Pol immunized serum samples did not always recognize the same epitopes as those recognized by native immunized serum samples but recognized other epitopes of the native allergens as shown in Figure 2.

Membrane was incubated with the pool of preimmune sera, and no peptide was recognized (data not shown).

### 3.3. IgG Inhibition

**Figure 3:**
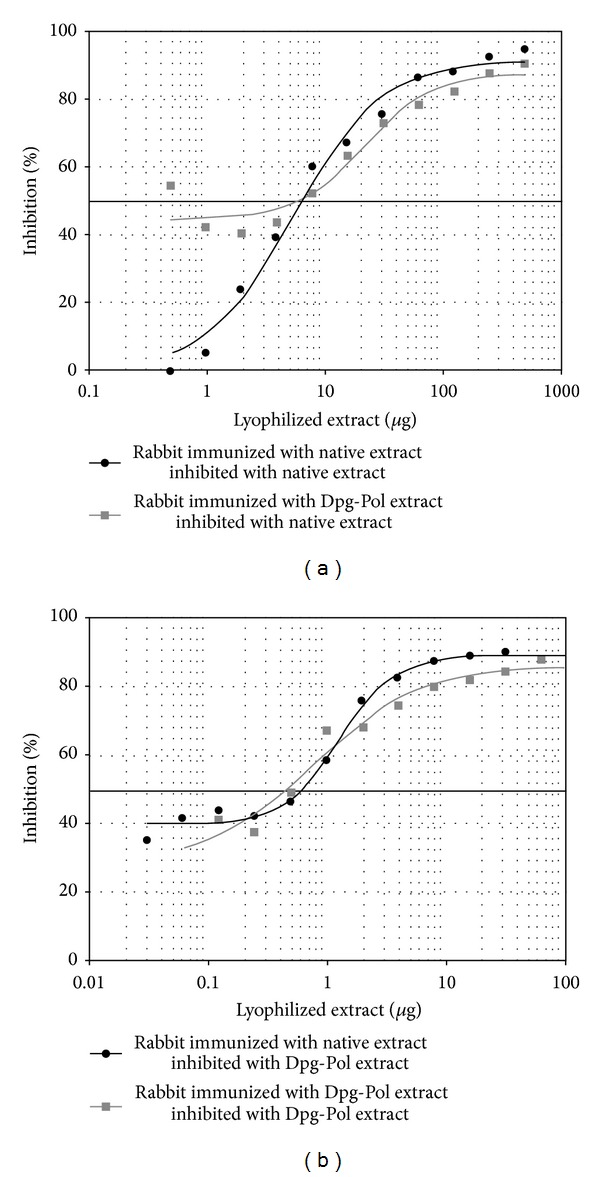
IgG inhibition. Graphics represent the percentage of inhibition for every serum with native or Dpg-Pol extract. (a) Native extract in the solid phase. (b) Dpg-Pol extract in the solid phase. Quantity of lyophilized extract used in the inhibition is in a logarithmic scale. 50% inhibition is marked with a thicker line.

Inhibition experiments using serum from native and Dpg-Pol immunized rabbits with native and polymerized extracts showed differences in the IgG response to the two extracts. When native extract was incubated with native immunized serum samples and inhibited itself, a 50% inhibition point of 7.96 *μ*g of protein was obtained, with a sigmoidal curve between 0% and 100% of inhibition. However, after incubation of native extract with Dpg-Pol immunized serum it was not possible to calculate the 50% inhibition, because the inhibition curve did not reach total inhibition (Figure 3(a)). Similar results were obtained when Dpg-Pol was used in solid phase (Figure 3(b)).

### 3.4. Immunoblot Inhibition

**Figure 4:**
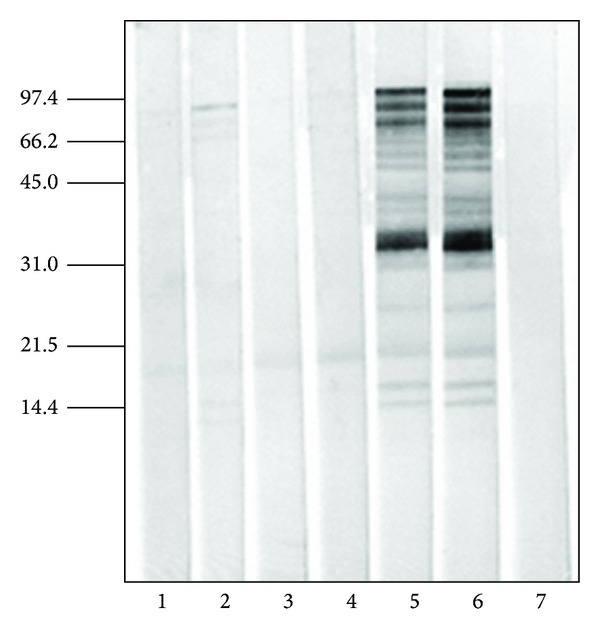
Immunoblot inhibition. *B. alba* native extract (65 *μ*g/line) in solid phase. Line 1: Rabbit 978 (immunized with native extract), line 2: Rabbit 979 (immunized with native extract), line 3: Rabbit 976 (immunized with Dpg-Pol extract), and line 4: Rabbit 977 (immunized with Dpg-Pol extract); lines 1 to 4 were inhibited with native extract (650 *μ*g of *B. alba* native extract). Line 5: Rabbit 979 without inhibition, line 6: Rabbit 979 inhibited with BSA, and line 7: negative control (PBST+5% milk). Sera were used at a dilution 1 : 400.

When native extract was used in solid phase, serum samples from native and Dpg-Pol immunized animals were capable of inhibiting practically the 100% of the extract (Figure 4, lines 1 to 4) confirming that the IgG antibodies synthesized during the immunization process are being produced against the antigens present in the allergenic source. Noninhibited controls recognized all the antigens present in native extracts (Figure 4, lines 5 and 6).

### 3.5. IgE Binding Sites Blocking Antibodies

When native extract and purified Bet v 1 were inhibited with serum samples from native and Dpg-Pol immunized animals and afterwards incubated with the pool of human sera, the results demonstrated that human IgE binding epitopes of the native extract and those of rBet v 1 were blocked by rabbit IgG antibodies induced by both native and Dpg-Pol extract. A curve of inhibition between 0% and 100% was obtained. 

**Figure 5:**
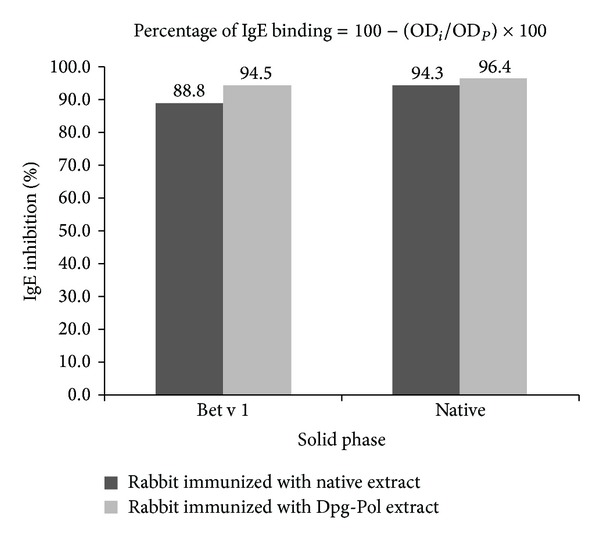
Inhibition of human IgE antibodies with the IgG produced in immunized rabbits (dilution 1/2). Native extract of *B. alba* or rBet v 1 is used in solid phase. The human pool of sera was diluted to 1/10. The formula for calculating the percentage of IgE inhibition is shown, OD_*i*_ and OD_*P*_ being the optical densities after preincubation with the rabbit's immune sera and the corresponding preimmune sera, respectively.

The percentage of IgE inhibition was calculated according to the formula previously described. For a rabbit sera dilution of 1 : 2 serum samples with native induced antibodies inhibited IgE binding to the whole extract by 88.8% while serum samples with Dpg-Pol induced antibodies inhibited by 94.5%. IgG induced by native and Dpg-Pol extracts inhibited IgE binding to Bet v 1 by 94.3 and 96.4%, respectively (Figure 5).

## 4. Discussion

The clinical efficacy of allergen immunotherapy has been related to induction of IgG antibodies that block IgE-allergen interaction [2]. The ability to elicit specific IgG, and specially IgG4, antibodies by allergenic vaccines against the components of these extracts has been demonstrated in different published studies [11]. Here we show that Dpg-Pol birch pollen extract induced IgG antibodies to a range of allergen epitopes from Bet v 1 and Bet v 2 and that these IgG antibodies inhibited binding of human IgE to birch pollen allergenic extract. These findings suggest that induction of blocking IgG antibodies may also play a part in the clinical efficacy of Dpg-Pol vaccines.

In general terms it is accepted that exogenous antigens are captured by antigen presenting cells, processed in small peptides, combined with MHC class II molecules, and finally presented to different cells [19]. However, allergoids and Dpg-Pol molecules have different structure, size, and characteristics [14], and how they are handled by antigen-presenting cells is unknown yet. We have previously shown reduced activation of effector T cells by Dpg-Pol extracts compared to native allergen extracts but conserved activity of regulatory T cells [20]. Here we confirm that Dpg-Pol extracts induce IgG antibody response *in vivo*, broadly similar to that for native allergen extract. However, the polymerization with glutaraldehyde may also modify the tertiary and quaternary structure of the molecules as we also observed recognition of specific IgG antibodies to new regions or epitopes in the individual allergens. Besides, the polymerization reaction modifies conformational epitopes that represent the majority of allergen IgE epitopes [5], and as has been previously demonstrated, Dpg-Pol extracts have greatly reduced IgE binding capacity *in vivo* and *in vitro* [20, 21] compared to native extracts. Depigmentation-polymerization process synthesized new antigens consisting of allergen chains with new epitopes, which have the capacity to stimulate the induction of specific IgG not present after immunization with native molecules, blocking new regions that native extracts are not able to block.

According to these concepts of creation of new structures with new IgG epitopes after polymerization, ELISA inhibition experiments have always shown different curves when native and Dpg-Pol extracts are compared. In our study, comparing the sigmoidal curves obtained using serum from native or Dpg-Pol immunized animals, we observed a different inhibition pattern capacity when they were used with native or Dpg-Pol extracts in solid phase or as inhibitors. That means that the induced antibodies are recognising different structures in the molecules, although in both cases the inhibition capacity was comparable and correlates perfectly when each serum sample is inhibited with its corresponding inducing extract. But, though the formation of new epitopes with capacity to stimulate new specific antibodies is important for the improvement of the immunological effect from an allergic point of view, the real benefit of these new antibodies is their capacity to block IgE epitopes, where IgE is binding to allergens inducing the allergenic response. The capacity of IgG induced by both Dpg-Pol and native birch pollen extract to block human IgE-allergen interaction was demonstrated in our study by combining human and rabbit samples following the method previously described by Ball et al. [18]. IgE binding was almost totally inhibited by IgG induced by both extracts, and this inhibition was concentration dependent as previous studies in humans demonstrated [22]. The consideration of these aspects in the development of new vaccines should be taken into account and seems to be interesting [23]. 

In this study we used experimental animals because they have not previously been exposed to birch pollen. It would be of interest to study epitope specificity of IgG4 induced in patients given immunotherapy with Dpg-Pol extracts, but this would be more complex because of varied immune response and the presence of IgG antibodies before treatment. We have previously demonstrated increases in allergen-specific IgG4 in patients treated with Dpg-Pol extracts [11], and we are currently examining the IgE blocking activity of such IgG4 antibodies.

In summary, mapping peptides can be a potent tool for the identification of specific regions related to the immunological response of allergenic vaccines. According to our results, depigmented-polymerized allergenic molecules acquire a different structure, from an immunological point of view, stimulating the synthesis of most of the IgG induced by native extracts and also specific antibodies, which are not induced by native extracts, with capacity to recognized new linear sequences in Bet v 1 and Bet v 2. This immunological response produces blocking antibodies which not only have capacity to inhibit IgG antibodies produced after the immunization with native extracts, but also are able to block specific regions of IgE binding sites in allergens. These results explain and are in agreement with the presence of blocking antibodies demonstrated in clinical trials.

## Figures and Tables

**Table 1:** Synthetic peptides of Bet v 1 and Bet v 2. They are synthetized overlapping by 6 aminoacids; their position in the sequence, and the recognition by the IgG induced after the immunization with Dpg-Pol (D-P) or native (N) extracts are shown.

Number	Sequence	Position	IgG extract recognition
Bet v 1			
1	MGVFNYETETTS	1–12	
2	ETETTSVIPAAR	7–18	D-P/N
3	VIPAARLFKAFI	13–24	D-P/N
4	LFKAFILDGDNL	19–30	N
5	LDGDNLFPKVAP	25–36	
6	FPKVAPQAISSV	31–42	D-P/N
7	QAISSVENIEGN	37–48	D-P
8	ENIEGNGGPGTI	43–54	D-P
9	GGPGTIKKISFP	49–60	N
10	KKISFPEGFPFK	55–66	N
11	EGFPFKYVKDRV	61–72	
12	YVKDRVDEVDHT	67–78	
13	DEVDHTNFKYNY	73–84	
14	NFKYNYSVIEGG	79–90	
15	SVIEGGPIGDTL	85–96	D-P/N
16	PIGDTLEKISNE	91–102	N
17	EKISNEIKIVAT	97–108	N
18	IKIVATPDGGSI	103–114	D-P
19	PDGGSILKISNK	109–120	D-P/N
20	LKISNKYHTKGD	115–126	
21	YHTKGDHEVKAE	121–132	
22	HEVKAEQVKASK	127–138	N
23	QVKASKEMGETL	133–144	
24	EMGETLLRAVES	139–150	
25	LRAVESYLLAHS	145–156	
26	YLLAHSDAYN	151–160	

Bet v 2			
1	MSWQTYVDEHLM	1–12	
2	YVDEHLMCDIDG	6–17	
3	LMCDIDGQASNS	11–22	
4	DGQASNSLASAI	16–27	
5	SLASAIVGHDGS	22–33	
6	AIVGHDGSVWAQ	26–37	
7	DGSVWAQSSSFP	31–42	
8	AQSSSFPQFKPQ	36–47	D-P
9	FPQFKPQEITGI	41–52	D-P/N
10	PQEITGIMKDFE	46–57	D-P/N
11	GIMKDFEEPGHL	51–62	
12	FEEPGHLAPTGL	56–67	D-P
13	HLAPTGLHLGGI	61–72	
14	GLHLGGIKYMVI	66–77	
15	GIKYMVIQGEAG	71–82	
16	VIQGEAGAVIRG	76–87	N
17	AGAVIRGKKGSG	81–92	D-P/N
18	RGKKGSGGITIK	86–97	D-P/N
19	SGGITIKKTGQA	91–102	D-P/N
20	IKKTGQALVFGI	96–107	D-P/N
21	ALVFGIYEEPVT	102–113	D-P
22	GIYEEPVTPGQC	106–117	
23	TPVTPGQCNMVV	110–122	
24	GQCNMVVERLGD	115–126	
25	VERLGDYLIDQGL	121–133	N
